# Anti-Inflammatory Activity of Synaptamide in the Peripheral Nervous System in a Model of Sciatic Nerve Injury

**DOI:** 10.3390/ijms24076273

**Published:** 2023-03-27

**Authors:** Anna Starinets, Anna Tyrtyshnaia, Igor Manzhulo

**Affiliations:** A.V. Zhirmunsky National Scientific Center of Marine Biology, Far Eastern Branch, Russian Academy of Sciences, 690041 Vladivostok, Russia; anan.star13@yandex.ru (A.S.); anna.tyrtyshnaia@bk.ru (A.T.)

**Keywords:** chronic constriction injury, N-docosahexaenoylethanolamine, synaptamide, sciatic nerve, dorsal root ganglia, microglia, astroglia

## Abstract

N-docosahexaenoylethanolamine (DHEA), or synaptamide, is an endogenous metabolite of docosahexaenoic acid (DHA) that exhibits synaptogenic and neurogenic effects. In our previous studies, synaptamide administration inhibited the neuropathic pain-like behavior and reduced inflammation in the central nervous system following sciatic nerve injury. In the present study, we examine the effect of synaptamide on the peripheral nervous system in a neuropathic pain condition. The dynamics of ionized calcium-binding adapter molecule 1 (iba-1), CD68, CD163, myelin basic protein, and the production of interleukin 1β and 6 within the sciatic nerve, as well as the neuro-glial index and the activity of iba-1, CD163, glial fibrillary acidic protein (GFAP), neuronal NO synthase (nNOS), substance P (SP), activating transcription factor 3 (ATF3) in the dorsal root ganglia (DRG), are studied. According to our results, synaptamide treatment (4 mg/kg/day) (1) decreases the weight-bearing deficit after nerve trauma; (2) enhances the remyelination process in the sciatic nerve; (3) shows anti-inflammatory properties in the peripheral nervous system; (4) decreases the neuro-glial index and GFAP immunoreactivity in the DRG; (5) inhibits nNOS- and SP-ergic activity in the DRG, which might contribute to neuropathic pain attenuation. In general, the current study demonstrates the complex effect of synaptamide on nerve injury, which indicates its high potential for neuropathic pain management.

## 1. Introduction

Currently, one of the most urgent topics for research in neuroscience is chronic pain management, in particular, the management of chronic neuropathic pain that is caused by injury to the peripheral nervous system and leads to the development and maintenance of many somatic and psychophysiological disorders [1,2].

Gamma-aminobutyric acid (GABA) analogs and inhibitors and tricyclic antidepressants are among the first-line treatment options for neuropathic pain syndrome [3,4], whereas second-line options include capsaicin [5] and lidocaine patches. Neuropathic pain treatment also includes strong opioids as third-line treatments and various interventional therapies, such as nerve blocks and surgery for targeted drug delivery [3,4]. However, at present, the treatment options for neuropathic pain syndrome are limited and not effective enough for pain attenuation [3,4].

Some of the recently developed alternative methods for neuropathic pain treatment include substances of natural origin, such as polyunsaturated fatty acids (PUFAs) and their metabolites, that can act as neuromodulators and anti-inflammatory compounds in both peripheral and central nervous systems, prevent cytokine secretion, and block the synthesis of prostaglandins, leukotrienes, and thromboxanes [6,7]. In particular, docosahexaenoic acid (DHA) is a PUFA that was used in multiple studies due to its anti-inflammatory and neuroprotective properties [7,8,9,10,11]. However, DHA is believed to be active mainly through its derivatives and metabolites [12]. As previously described, DHA is metabolized into highly active compounds, including resolvins, which actively facilitate the resolving phase of acute inflammation, unlike protein mediators that act during the onset phase of inflammation [13]. For instance, resolvin D1 has been shown to promote the targeting and clearance of necroptotic cells in murine plaques, the accumulation of which can be damaging to tissues [14]. Thus, resolvins are considered key compounds for the treatment of inflammation-related diseases [12].

Synaptamide is another endogenous metabolite of DHA that can promote neurite- and synaptogenesis in neuronal cell cultures [15,16,17,18] and differentiation in neural stem cells [17]. Moreover, synaptamide injection into pregnant mice induced neurogenesis in the brains of the offspring mice [19]. Synaptamide administration to microglial cell culture after lipopolysaccharide (LPS) treatment inhibits the release of proinflammatory cytokines [20,21]. Despite being a structural analog of anandamide (N-arachidonoylethanolamine), synaptamide exhibits weak binding to cannabinoid receptors, thus most likely acting in an endocannabinoid-independent manner. Synaptamide is synthesized from DHA in the brain and retina, possibly via the N-acylation phosphodiesterase pathway, similar to other N-acylethanolamines [18]. As recently shown, the neurogenic, neuritogenic, and synaptogenic properties of synaptamide are mediated by its target receptor, the G-protein-coupled receptor 110 (GPR110) [22,23]. Activation of GPR110/cAMP signaling by synaptamide binding with the N-terminal domain, particularly the conserved GAIN/GPS motif of GPR110, is leading to cAMP elevation in microglia and innate peripheral immune cells, which is essential for the anti-inflammatory effects of synaptamide [22]. In particular, activation of this receptor by synaptamide leads to a decrease in microglial activity and pro-inflammatory cytokine production [19]. Synaptamide targeting GPR110 is further proven by the absence of synaptamide-induced biological activity in GPR110 knockout mice, whereas overexpression of GPR110 leads to the enhancement of synaptamide activity [22].

We have previously discovered that synaptamide treatment after the chronic constriction injury of the sciatic nerve affects the neuropathic pain development in rats, resulting in significantly less pronounced pain-like behavior [24,25,26], including a decrease in cold allodynia [25,26], as well as thermal [24] and mechanical hyperalgesia [26]. Furthermore, daily synaptamide administration prevents increased anxiety, and the impairment of locomotor activity, working, and long-term memory, caused by neuropathic pain syndrome [24,25]. Synaptamide administration for 35 days after sciatic nerve injury reduces inflammation in the lumbar spinal cord [26] and the hippocampus [24] by decreasing microglia activity and proinflammatory cytokine concentrations. Synaptamide also improves neurogenesis in the dentate gyrus subgranular zone of the hippocampus 14- and 35-days post-injury [24,25]. as well as stabilizes astroglial activity and increases the expression of nerve growth factor (NGF) and two subunits of N-methyl-D-aspartate (NMDA) receptors in the hippocampus 14 days after nerve trauma [25]. Furthermore, synaptamide increases the concentration of N-acylethanolamines in plasma and the brain, in particular, the levels of oleoylethanolamide, palmitoylethanolamide, and arachidonylethanolamide [24,25]. Moreover, synaptamide administration results in the upregulation of superoxide dismutase (SOD) activity in microglia and astroglia cell cultures, thus improving the antioxidant activity and preventing peroxidative stress reactions [20,26]. The systemic anti-inflammatory and antioxidant properties of synaptamide are well-established features in the central nervous system (CNS); however, to date, the effects of synaptamide treatment on the peripheral nervous system have not been studied.

Thus, in our current research, we investigate the effects of synaptamide treatment on (1) neuropathic pain severity by evaluating the weight-bearing difference in vehicle- and synaptamide-treated rats following chronic constriction injury (CCI) of the sciatic nerve; (2) the regeneration process by assessing the remyelination in the injured nerve and activating transcription factor 3 (ATF3) activity in the primary sensory neurons in the dorsal root ganglia (DRG) by immunohistochemical studies; (3) inflammation in the sciatic nerve and DRG by immunohistochemical staining and enzyme-linked immunosorbent assay (ELISA); (4) satellite glial cells, neuronal NO-synthase (nNOS) and substance P (SP) activity in the DRG by immunohistochemical studies. We hypothesize that synaptamide treatment of neuropathic pain syndrome caused by sciatic nerve injury reduces inflammation in the peripheral nervous system, which results in downregulation of nNOS and SP activity, and thus, contributes to neuropathic pain attenuation.

## 2. Results

### 2.1. Weight-Bearing Difference Induced by Chronic Constriction Injury and Following Synaptamide Treatment

**Figure 1:**
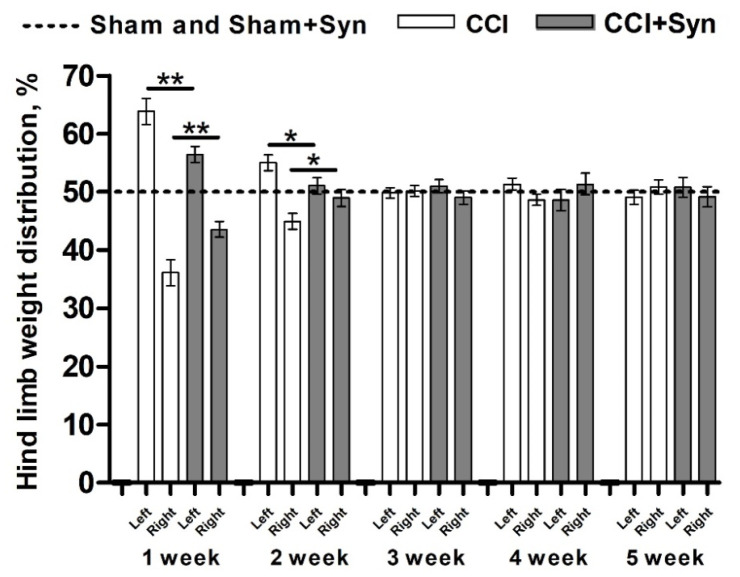
Hind limb weight distribution in neuropathic pain condition and following synaptamide treatment, mean ± SEM, n = 14 (number of animals/group) * *p* < 0.05, ** *p* < 0.01 (Mann-Whitney test). “Sham”—sham-operated animals that received vehicle treatment; “Sham + Syn”—sham-operated rats that underwent synaptamide administration; “CCI”—animals with chronic constriction injury of the sciatic nerve that received vehicle treatment; “CCI + Syn”—synaptamide-treated rats with chronic constriction injury of the sciatic nerve that underwent synaptamide treatment.

Vehicle-treated sham-operated rats (the “Sham” group) and synaptamide-treated sham-operated rats (the “Sham + Syn” group) symmetrically distributed their weight on their hind limbs throughout the experiment. At week 1 post-surgery, the weight distribution pattern was evaluated as 50.7 ± 1.4%:49.3 ± 1.4% between left and right limbs, respectively, in the “Sham” group and 49.1 ± 1.5%:50.9 ± 1.5% in the “Sham + Syn” group (Figure 1).

However, 1-week post-surgery, the weight distribution pattern for vehicle-treated rats with CCI of the sciatic nerve (the “CCI” group) changed drastically: 63.9 ± 2.2%:36.1 ± 2.2% between intact and injured limbs, respectively (Figure 1). However, at this time point, the weight distribution asymmetry was significantly less severe in synaptamide-treated rats with CCI of the sciatic nerve (the “CCI + Syn” group): 56.4 ± 1.4%:43.6 ± 1.4% (Figure 1).

At week 2, the asymmetry in weight distribution in the “CCI” group decreased to 55.0 ± 1.4%:45.0 ± 1.4%; whereas rats in the “CCI + Syn” group demonstrated almost symmetrical weight distribution between intact and injured limbs: 51.0 ± 1.4%:49.0 ± 1.4% (Figure 1). In the sham-operated rats, the weight distribution pattern was estimated as 49.8 ± 1.9%:50.2 ± 1.9% between left and right limbs, respectively, in the “Sham” group, and 49.5 ± 1.7%:50.5 ± 1.7% in the “Sham + Syn” group (Figure 1).

At week 3 and up to the end of the experiment, the weight distribution was almost equal in both the “CCI” and “CCI + Syn” groups (Figure 1). In particular, at week 3, the weight distribution pattern was evaluated as 49.8 ± 0.9%:50.2 ± 0.9% in the “CCI” group and 51.0 ± 1.1%:49.0 ± 1.1% in the “CCI + Syn” group. At week 4, the weight distribution pattern was 51.4 ± 1.0%:48.6 ± 1.0% in the “CCI” group and 48.6 ± 1.8%:51.4 ± 1.8% in the “CCI + Syn” group. At week 5, the weight distribution pattern was evaluated as 49.2 ± 1.2%:50.8 ± 1.2% in the “CCI” group and 50.8 ± 1.7%:49.2 ± 1.7% in the “CCI + Syn” group.

### 2.2. Synaptamide Increases Remyelination after Chronic Constriction Injury

**Figure 2:**
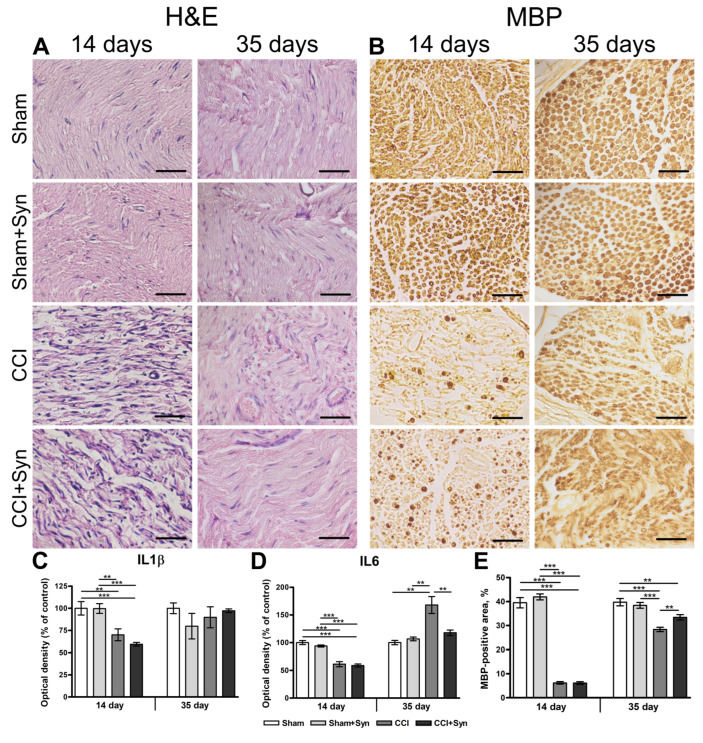
(**A**) The distal segment of the sciatic nerve after chronic constriction injury and following synaptamide treatment (hematoxylin–eosin staining). (**B**) Myelin basic protein (MBP) distribution in the distal segment of the sciatic nerve. Scale bar is 50 µm. (**C**) Optical density (% of control) of interleukin-1 beta (IL-1β) in the sciatic nerve, mean ± SEM, n = 7 (number of animals/group) ** *p* < 0.01, *** *p* < 0.001 (one-way ANOVA, post-test Tukey). (**D**) Optical density (% of control) of interleukin-6 (IL-6) in the sciatic nerve, mean ± SEM, n = 7 (number of animals/group). (**E**) The staining area (% of total area of the nerve) of MBP-positive structures in the sciatic nerve, mean ± SEM, n = 7 (number of animals/group) ** *p* < 0.01, *** *p* < 0.001 (one-way ANOVA, post-test Tukey). “Sham”—sham-operated animals that received vehicle treatment; “Sham + Syn”—sham-operated rats that underwent synaptamide administration; “CCI”—animals with chronic constriction injury of the sciatic nerve that received vehicle treatment; “CCI + Syn”—synaptamide-treated rats with chronic constriction injury of the sciatic nerve that underwent synaptamide treatment.

Chronic constriction injury of the sciatic nerve was followed by swelling and derangement of the nerve fibers distal to the site of trauma [27], which can be observed via hematoxylin-eosin staining on day 14 post-surgery in both the “CCI” and “CCI + Syn” groups. At day 35, nerve structure is almost recovered in both synaptamide- and vehicle-treated rats (Figure 2A). 

CCI leads to demyelination and degradation of myelin sheaths as a consequence of axonal degeneration distal to the injury and can be observed by the decrease in expression of myelin markers in immunohistochemical staining [27]. Myelin sheaths of the nerve fibers also underwent significant changes 14 days after sciatic nerve injury, which can be detected by myelin basic protein (MBP) staining (Figure 2B). While in the “Sham” and “Sham + Syn” groups the area fraction of MBP immunostaining occupied 39.5 ± 2.2% and 41.9 ± 1.2% of the sciatic nerve, respectively, the staining area of MBP-positive structures was drastically reduced to 6.2 ± 0.5% in the “CCI” group (*p* < 0.001), and 6.1 ± 0.5% in the “CCI + Syn” group (*p* < 0.001) (Figure 2B,E). However, 35 days post-surgery, MBP immunoreactivity increased to 33.5 ± 1.1% in synaptamide-treated rats, which was significantly higher in comparison to the “CCI” group (28.4 ± 0.9%, *p* < 0.01) (Figure 2B,E). Nevertheless, MBP-positive structures were not completely restored in the “CCI + Syn” group, and their staining area remained significantly lower than in the “Sham” (39.7 ± 1.5%, *p* < 0.01) and “Sham + Syn” groups (38.4 ± 1.2%, *p* < 0.001) (Figure 2B,E).

### 2.3. Synaptamide Treatment Inhibits Inflammation in the Sciatic Nerve after Injury

**Figure 3:**
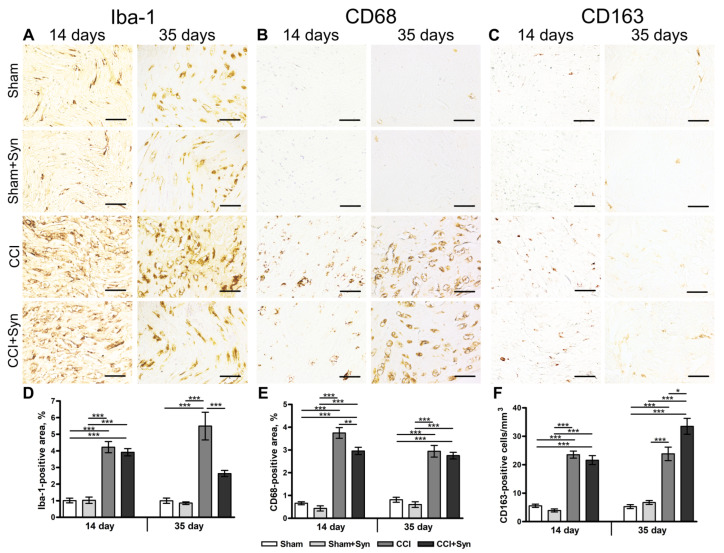
Inflammation in the distal segment of the sciatic nerve. Representative images of (**A**) ionized calcium-binding adapter molecule 1- (iba-1-), (**B**) CD68- and (**C**) CD163-positive staining. Scale bar is 50 µm. (**D**) The staining area (% of total area of the nerve) of iba-1-positive macrophages within the distal segment of the sciatic nerve, mean ± SEM, n = 7 (number of animals/group) *** *p* < 0.001 (one-way ANOVA, post-test Tukey). (**E**) The staining area (% of total area of the nerve) of CD68-positive macrophages within the distal segment of the sciatic nerve, mean ± SEM, n = 7 (number of animals/group) ** *p* < 0.01, *** *p* < 0.001 (one-way ANOVA, post-test Tukey). (**F**) The staining area (% of total area of the nerve) of CD163-positive macrophages within the distal segment of the sciatic nerve, mean ± SEM, n = 7 (number of animals/group) * *p* < 0.05, *** *p* < 0.001 (one-way ANOVA, post-test Tukey). “Sham”—sham-operated animals that received vehicle treatment; “Sham + Syn”—sham-operated rats that underwent synaptamide administration; “CCI”—animals with chronic constriction injury of the sciatic nerve that received vehicle treatment; “CCI + Syn”—synaptamide-treated rats with chronic constriction injury of the sciatic nerve that underwent synaptamide treatment.

Axonal regeneration in damaged nerves is accompanied by the inflammatory process, which involves the activation, proliferation, and migration of macrophages to the site of injury [27,28]. In the current research, sciatic nerve injury resulted in local inflammation that was evident 14 days post-surgery (Figure 3A–C).

The increase in the amount of ionized calcium-binding adapter-1 (iba-1) is considered the morpho-chemical indicator of macrophage activity upregulation [28]. Increased activity of iba-1-positive macrophages was observed in the nerve tissue in both groups with nerve injury compared to “Sham” (1.0 ± 0.1%, *p* < 0.001) and “Sham + Syn” (1.0 ± 0.2%, *p* < 0.001) groups (Figure 3A,D). No significant difference was detected between the “CCI” (4.2 ± 0.3%) and “CCI + Syn” (3.9 ± 0.2%) groups at that time point (Figure 3D). However, at day 35 after injury, synaptamide administration resulted in a significant downregulation of iba-1 immunoreactivity in the “CCI + Syn” group (2.6 ± 0.2%), in comparison to the “CCI” group (5.5 ± 0.8%, *p* < 0.001) (Figure 3A,D). Nevertheless, the iba-1-positive staining area in the “CCI + Syn” group was still upregulated in comparison to that of the sham-operated rats (1.0 ± 0.2%, *p* < 0.001 in “Sham”, and 0.9 ± 0.1%, *p* < 0.001 in “Sham + Syn”) (Figure 3A,D).

CD163 is a member of the scavenger receptor cysteine-rich family and is expressed by anti-inflammatory M2 macrophages, which produce anti-inflammatory cytokines and opioid peptides that are important for neuropathic pain reduction [29,30]. At day 14 after the surgery, CD163 immunoreactivity was significantly increased in both groups that underwent the chronic constriction injury, in comparison to the “Sham” (5.6 ± 0.6%, *p* < 0.001) and “Sham + Syn” (4.0 ± 0.5%, *p* < 0.001) groups (Figure 3C,F). At the time point, the statistical analysis did not detect a significant difference between the “CCI” (23.6 ± 1.2%) and “CCI + Syn” (21.6 ± 1.6%) groups (Figure 3F). However, 35 days post-surgery, synaptamide administration caused a significant increase in CD163-positive staining in the “CCI + Syn” group (33.5 ± 2.8%), compared to the “CCI” group (23.8 ± 2.4%, *p* < 0.05) (Figure 3C,F). At the same time, CD163 immunoreactivity in sham-operated rats was markedly lower than in both groups with nerve injury (5.3 ± 0.7%, *p* < 0.001, in “Sham”, and 6.7 ± 0.7%, *p* < 0.001, in “Sham + Syn”) (Figure 3C,F).

CD68 is a surface marker of the antigen-activated proinflammatory M1 macrophages [30], which express proinflammatory molecules, such as tumor necrosis factor α (TNF-α), interleukin 6 (IL-6), and interferon-gamma (IFN-γ) [29], which are essential for tissue protection, but may also result in tissue damage [31]. At day 14 after surgery, an elevated immunoreactivity of CD68-positive macrophages was observed in the sciatic nerve tissue in both groups with chronic constriction injury in comparison to the “Sham” (0.7 ± 0.3%, *p* < 0.001) and “Sham + Syn” (0.4 ± 0.1%, *p* < 0.001) groups (Figure 3B,E). However, synaptamide treatment resulted in a significant decrease in CD68-positive macrophage activity in the “CCI + Syn” group (3.0 ± 0.2%), in comparison to the “CCI” group (3.8 ± 0.2%, *p* < 0.01) (Figure 3B,E). At day 35 after surgery, CD68 immunoreactivity was still increased in the “CCI” (3.0 ± 0.3%) and “CCI + Syn” (2.8 ± 0.1%) groups, compared to the “Sham” (0.8 ± 0.1%, *p* < 0.001) and “Sham + Syn” (0.6 ± 0.1%, *p* < 0.001) groups (Figure 3B,E). However, statistical analysis did not reveal a significant difference between the “CCI” and “CCI + Syn” groups at this time point (Figure 3E).

IL-1β is a proinflammatory cytokine that is upregulated in the immature Schwann cells in the early stage of degeneration following peripheral nerve trauma distal to the injury site. However, IL-1β expression decreases after the remyelination process begins [32]. ELISA of the interleukins in the sciatic nerve detected a decrease in the IL-1β production in the “CCI” (70.1 ± 6.9%, *p* < 0.01) and “CCI + Syn” groups (59.3 ± 2.1%, *p* < 0.001), compared to the “Sham” (100 ± 7.6%) and “Sham + Syn” (99.8 ± 5.5%) groups at day 14 post-surgery (Figure 2C). However, a significant difference in the IL-1β levels between the experimental groups was not registered 35 days after surgery (Figure 2C). 

IL-6 is a multifunctional cytokine that is upregulated in the nervous system in pathological conditions, such as injury and inflammatory stimulus [33]. In the case of sciatic nerve injury, IL-6 levels increase distally from the site of injury [34] and in the large and medium lumbar DRG neurons [35]. At day 14 after surgery, IL-6 production was significantly decreased in both “CCI” (61.3 ± 4.5%) and “CCI + Syn” (58.9 ± 2.9%) groups, in comparison to “Sham” (100.0 ± 3.6%, *p* < 0.001) and “Sham + Syn” (93.9 ± 1.9%, *p* < 0.001) groups (Figure 2D). However, a significant increase in IL-6 level was detected in vehicle-treated rats with CCI 35 days post-surgery (168 ± 15.3%, *p* < 0.01) (Figure 2D). The optical density of IL-6 was significantly lower in synaptamide-treated animals (117.8 ± 5% of control) than in the “CCI” group (*p* < 0.01) (Figure 2D). Notably, the IL-6 level in the “CCI + Syn” group did not significantly differ from the IL-6 levels of both the “Sham” (100 ± 4.0%) and “Sham + Syn” (106.6 ± 3.4%) groups (Figure 2D).

### 2.4. Synaptamide Affects the Neuro-Glial Index in the Dorsal Root Ganglia

**Figure 4:**
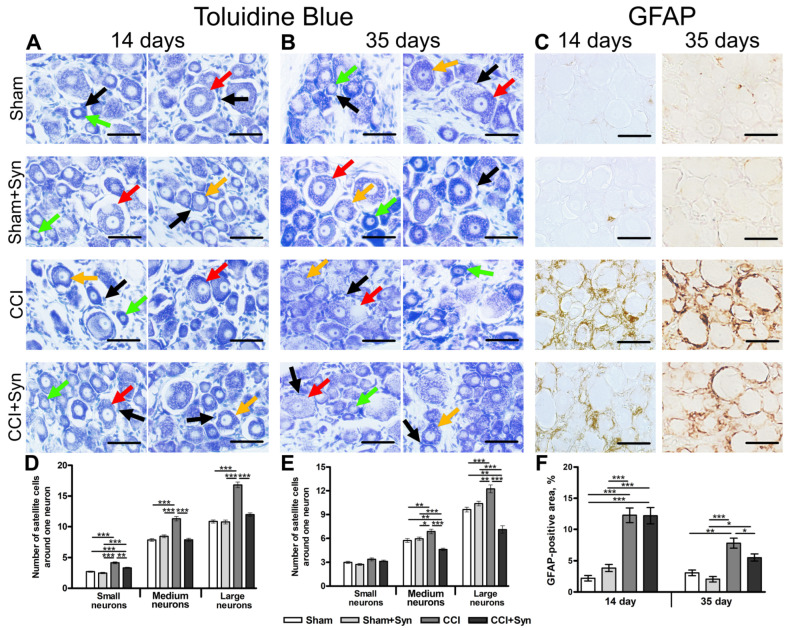
Neuro-glial index in the ipsilateral dorsal root ganglia (DRG). Representative images of (**A**,**B**) satellite glial cells (black arrows) around small (green arrows), medium-sized (orange arrows), and large (red arrows) neurons, (**C**) glial fibrillary acidic protein-(GFAP-) positive cells. Scale bar is 50 µm. (**D**) Neuro-glial index in the ipsilateral DRG 14 days post-surgery, mean ± SEM, n = 7 (number of animals/group) *** *p* < 0.001 (one-way ANOVA, post-test Tukey). (**E**) Neuro-glial index in the ipsilateral DRG 35 days post-surgery, mean ± SEM, n = 7 (number of animals/group) * *p* < 0.05, ** *p* < 0.01, *** *p* < 0.001 (one-way ANOVA, post-test Tukey). (**F**) The staining area (% of total area of the DRG) of GFAP-positive cells within the DRG, mean ± SEM, n = 7 (number of animals/group) * *p* < 0.05, ** *p* < 0.01, *** *p* < 0.001 (one-way ANOVA, post-test Tukey). “Sham”—sham-operated animals that received vehicle treatment; “Sham + Syn”—sham-operated rats that underwent synaptamide administration; “CCI”—animals with chronic constriction injury of the sciatic nerve that received vehicle treatment; “CCI + Syn”—synaptamide-treated rats with chronic constriction injury of the sciatic nerve that underwent synaptamide treatment.

Satellite glial cells (SGCs) form a distinct functional and morphological unit with each sensory neuron within the dorsal root ganglia. SGCs perform ion sink, insulation, and neurotransmitter recycling, and also buffer the extracellular environment via potassium and calcium channels. After peripheral nerve injury, SGCs undergo morphological and molecular alterations, including proliferation and an increase in coupling via gap junctions [36,37]. The number of SGCs around one neuron was significantly increased for small (4.2 ± 0.2), medium-sized (11.3 ± 0.4), and large (16.8 ± 0.5) neurons in the DRG after the CCI in vehicle-treated rats at day 14 post-surgery (Figure 4A,D). However, synaptamide administration prevented the increase in SGCs number in the “CCI + Syn” group, compared to the “CCI” group; the number of SGCs was estimated as 3.3 ± 0.1 (*p* < 0.01) for small, 7.9 ± 0.2 (*p* < 0.001) for medium-sized, and 12.0 ± 0.3 (*p* < 0.001) for large neurons (Figure 4D). Notably, no significant difference in the neuro-glial index of medium and large neurons was observed between the “CCI + Syn” group and both the “Sham” and “Sham + Syn” groups (Figure 4D). The number of SGCs around one neuron in the “Sham” group was measured as 2.7 ± 0.1, 7.9 ± 0.2, and 10.9 ± 0.3 for small, medium-sized, and large neurons, respectively (Figure 4A,D). At the same time, the neuro-glial index in the “Sham + Syn” group was almost identical to that of the “Sham” group and was estimated as 2.5 ± 0.1, 8.4 ± 0.2, and 10.8 ± 0.3 for small, medium-sized, and large neurons, respectively (Figure 4A,D). 

Moreover, 35 days after surgery, the number of SGCs around one small neuron did not differ significantly between “Sham” (3.0 ± 0.1), “Sham + Syn” (2.7 ± 0.1), “CCI” (3.4 ± 0.2), and “CCI + Syn” (3.1 ± 0.2) (Figure 4E). The neuro-glial index for medium-sized neurons was markedly increased in the “CCI” (6.9 ± 0.3) group, compared to the “CCI + Syn” (4.6 ± 0.2, *p* < 0.001), “Sham” (5.7 ± 0.2, *p* < 0.01), and “Sham + Syn” (5.9 ± 0.2, *p* < 0.05) groups (Figure 4B,E). Interestingly, the number of SGCs around one medium-sized neuron in the DRG of synaptamide-treated rats with CCI was significantly lower, than in both sham-operated groups (*p* < 0.01 and *p* < 0.001 for “Sham” and “Sham + Syn”, respectively) (Figure 4B,E). A similar pattern was observed for the number of SGCs around the large neurons 35 days after surgery. Neuro-glial index was significantly increased after injury in the “CCI” (12.2 ± 0.5) group, in comparison to the “Sham” (9.6 ± 0.3, *p* < 0.001) and “Sham + Syn” (10.4 ± 0.5, *p* < 0.01) (Figure 4B,E). However, the number of SGCs around one large neuron was markedly decreased following synaptamide administration in the “CCI + Syn” (7.1 ± 0.5) group, in comparison to vehicle-treated rats with CCI (*p* < 0.001) and both sham-operated groups (*p* < 0.001) (Figure 4B,E).

Glial fibrillary acidic protein is an intermediate filament protein, and its expression increases in SGCs in the DRG after sciatic nerve injury, which can be observed via immunohistochemistry [36]. At day 14 after surgery, the staining area of GFAP in the DRG was estimated as 2.2 ± 0.4% in the “Sham” group and 3.9 ± 0.6% in the “Sham + Syn” group (Figure 4C,F). Peripheral nerve injury in the “CCI” and “CCI + Syn” groups was accompanied by a pronounced upregulation in GFAP-positive staining up to 12.8 ± 1.2% and 12.2 ± 1.3%, respectively, compared to “Sham” (*p* < 0.001) and “Sham + Syn” (*p* < 0.001) (Figure 4C,F). No significant difference between the synaptamide- and vehicle-treated animals was present at this time point (Figure 4F). However, at day 35 after CCI, synaptamide treatment inhibited the dramatic increase in GFAP immunoreactivity (5.5 ± 0.6%, *p* < 0.05) in the “CCI + Syn” group, in comparison to the “CCI” group (7.8 ± 0.8%) (Figure 4C,F). GFAP immunostaining in the DRG of sham-operated rats was estimated as 3.1 ± 0.5% in the “Sham” group and 2.1 ± 0.4% in the “Sham + Syn” group (Figure 4C,F).

### 2.5. Synaptamide Decreases Inflammation in Dorsal Root Ganglia after Chronic Constriction Injury

**Figure 5:**
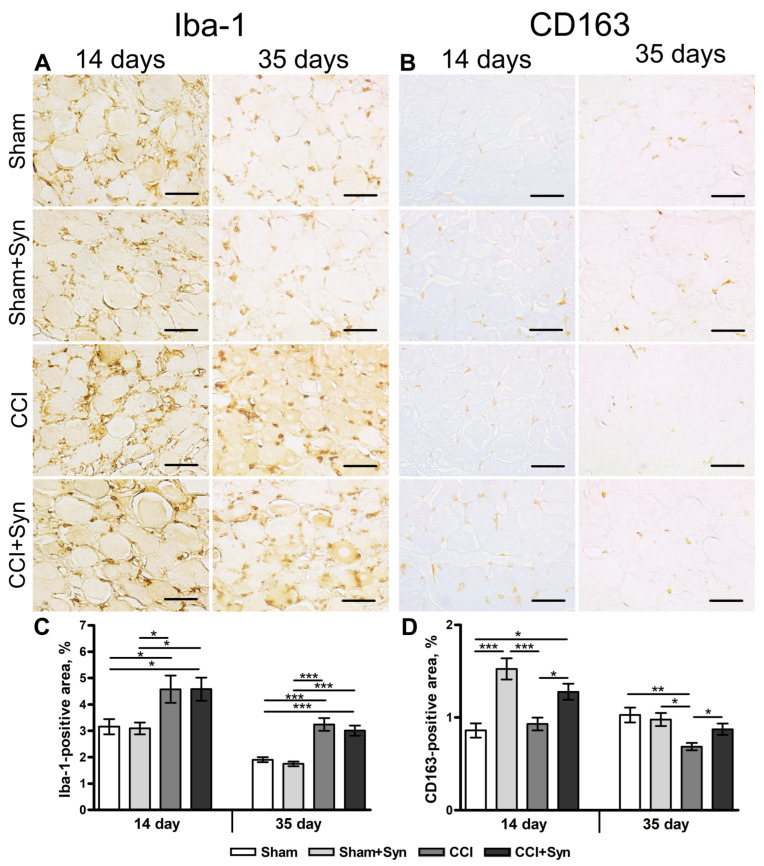
Inflammation in DRG after chronic constriction injury and synaptamide administration. Representative images of (**A**) iba-1-, (**B**) CD163-positive microglia/macrophages. Scale bar is 50 µm. (**C**) The staining area (% of total area of the DRG) of iba-1-positive microglia/macrophages within the DRG, mean ± SEM, n = 7 (number of animals/group) * *p* < 0.05, *** *p* < 0.001 (one-way ANOVA, post-test Tukey). (**D**) The staining area (% of total area of the DRG) of CD163-positive microglia/macrophages within the DRG, mean ± SEM, n = 7 (number of animals/group) * *p* < 0.05, ** *p* < 0.01, *** *p* < 0.001 (one-way ANOVA, post-test Tukey). “Sham”—sham-operated animals that received vehicle treatment; “Sham + Syn”—sham-operated rats that underwent synaptamide administration; “CCI”—animals with chronic constriction injury of the sciatic nerve that received vehicle treatment; “CCI + Syn”—synaptamide-treated rats with chronic constriction injury of the sciatic nerve that underwent synaptamide treatment.

Evaluation of the iba-1 immunostaining 14 days post-surgery showed an increase in microglia/macrophages activity in the DRG in both “CCI” (4.6 ± 0.5%) and “CCI + Syn” (4.6 ± 0.4%) groups in comparison to the “Sham” (3.2 ± 0.3%, *p* < 0.05) and “Sham + Syn” (3.1 ± 0.2%, *p* < 0.05) groups (Figure 5A,C). At day 35 after surgery, iba-1 immunoreactivity was still significantly upregulated in the ipsilateral DRG in both the “CCI” (3.2 ± 0.2%) and “CCI + Syn” (3.0 ± 0.2%) groups, in comparison to the “Sham” (1.9 ± 0.1%, *p* < 0.001) and “Sham + Syn” (1.8 ± 0.1%, *p* < 0.001) groups (Figure 5A,C).

Synaptamide treatment after the chronic constriction injury resulted in a significant increase in the immunoreactivity of the CD163-positive microglia/macrophages in both “CCI + Syn” (1.3 ± 0.1%) and “Sham + Syn” (1.5 ± 0.1%) groups 14 days post-surgery (Figure 5B,D). CD163-positive staining area was significantly lower in the vehicle-treated (0.9 ± 0.1%) than in the synaptamide-treated sham-operated rats (*p* < 0.001) (Figure 5D). Also, CD163 immunoreactivity in the “CCI” (1.5 ± 0.1%) group was markedly lower than in the “CCI + Syn” group (*p* < 0.05) (Figure 5B,D). Interestingly, no significant difference was detected between the “Sham” and “CCI” groups, as well as between the “Sham + Syn” and “CCI + Syn” groups on day 14 (Figure 5D). However, 35 days after the surgical procedure, CD163 immunoreactivity did not differ significantly between the “Sham” (1.0 ± 0.1%), “Sham + Syn” (1.0 ± 0.1%), and “CCI + Syn” groups (0.9 ± 0.1%) (Figure 5D). The CD163-positive staining area remained markedly lower in the “CCI” group (0.7 ± 0.03%) than in the “Sham” (*p* < 0.01), “Sham + Syn” (*p* < 0.05), and “CCI + Syn” (*p* < 0.05) groups (Figure 5B,D).

### 2.6. Synaptamide Affects Neurotransmitter Systems in Dorsal Root Ganglia after Chronic Constriction Injury

**Figure 6:**
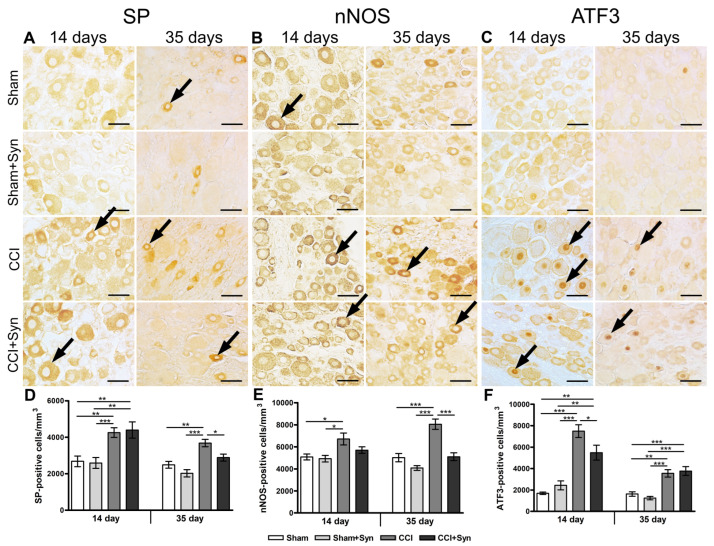
Representative images of ((**A**), black arrows) substance P- (SP-), ((**B**), black arrows) neuronal NO-synthase- (nNOS-), and ((**C**), black arrows) activating transcription factor 3- (ATF3-) positive neurons in the DRG. Scale bar is 50 µm. (**D**) The number of SP-positive neurons within the DRG, mean ± SEM, n = 7 (number of animals/group) * *p* < 0.05, ** *p* < 0.01, *** *p* < 0.001 (one-way ANOVA, post-test Tukey). (**E**) The number of nNOS-positive neurons within the DRG, mean ± SEM, n = 7 (number of animals/group) * *p* < 0.05, *** *p* < 0.001 (one-way ANOVA, post-test Tukey). (**F**) The number of ATF3-positive neurons within the DRG, mean ± SEM, n = 7 (number of animals/group) * *p* < 0.05, ** *p* < 0.01, *** *p* < 0.001 (one-way ANOVA, post-test Tukey). “Sham”—sham-operated animals that received vehicle treatment; “Sham + Syn”—sham-operated rats that underwent synaptamide administration; “CCI”—animals with chronic constriction injury of the sciatic nerve that received vehicle treatment; “CCI + Syn”—synaptamide-treated rats with chronic constriction injury of the sciatic nerve that underwent synaptamide treatment.

As previously suggested, the determinative way of pain attenuation after peripheral nerve injury is the modulation of substance P production in both the central and peripheral nervous systems [38]. Hence, changes in SP levels in the ipsilateral DRG were examined in our current study. On day 14 after surgery, the number of SP-positive neurons in the DRG was 2683 ± 289 cells per mm^3^ in the “Sham” group and 2595 ± 295 cells per mm^3^ in the “Sham + Syn” group (Figure 6D). Nerve injury in the “CCI” and “CCI + Syn” groups was accompanied by a pronounced increase in the number of SP-positive neurons up to 4257 ± 267 and 4400 ± 442 cells per mm^3^, respectively, in comparison to “Sham” (*p* < 0.01) and “Sham + Syn” (*p* < 0.001) (Figure 6A,D). At day 35, the number of neurons expressing SP in the DRG was 2492 ± 186 cells per mm^3^ in the “Sham” group and 2027 ± 200 cells per mm^3^ in the “Sham + Syn” group (Figure 6A,D). Chronic constriction injury caused a significant increase in the number of SP-positive neurons up to 3685 ± 201 cells per mm^3^ in vehicle-treated rats in comparison to the “Sham” (*p* < 0.01) and “Sham + Syn” (*p* < 0.001) groups (Figure 6A,D). However, synaptamide administration prevented an increase in the SP-positive neurons number (2886 ± 189 cells per mm^3^, *p* < 0.05) (Figure 6A,D).

NO acts as a neurotransmitter or intercellular messenger; however, in high concentrations, NO exhibits cytotoxicity and contributes to neuropathic pain manifestations, such as hyperalgesia. Furthermore, the overproduction of NO by the neuronal nitric oxide synthase (nNOS) is considered to be one of the fundamental causes of neuropathic pain [39]. At day 14 after surgery, the number of nNOS-positive neurons in the ipsilateral dorsal root ganglia was 5079 ± 277 cells per mm^3^ in the “Sham” group and 4921 ± 302 cells per mm^3^ in the “Sham + Syn” group (Figure 6E). Peripheral nerve injury in the “CCI” group was accompanied by a pronounced elevation of nNOS-positive neurons up to 6708 ± 540 cells per mm^3^ in comparison to “Sham” (*p* < 0.05) and “Sham + Syn” (*p* < 0.05) (Figure 6B,E). Following synaptamide administration, the number of nNOS-positive neurons was 5699 ± 296 cells per mm^3^ in the “CCI + Syn” group at 14 days after surgery (Figure 6B,E). However, the number of nNOS-positive neurons did not differ significantly between the “CCI + Syn” and “CCI” groups at this time point (Figure 6E). Nevertheless, at day 35 post-surgery, synaptamide treatment resulted in a pronounced decrease in the number of nNOS-positive neurons in the “CCI + Syn” group (5098 ± 356 cells per mm^3^), in comparison to the “CCI” group (8049 ± 473 cells per mm^3^, *p* < 0.001) (Figure 6B,E). Notably, the number of nNOS-positive neurons in the DRG of the “CCI + Syn” group did not differ significantly from both the “Sham” (5018 ± 382 cells per mm^3^) and “Sham + Syn” (4081 ± 218 cells per mm^3)^ groups (Figure 6B,E).

Moreover, synaptamide treatment had a significant effect on activating transcription factor 3 immunoreactivity in the ipsilateral DRG. ATF3 is an injury-related pro-regenerative transcription factor that stimulates nerve regeneration by activating the intrinsic growth state in injured neurons [40]. At day 14 after the surgical procedure, the number of ATF3-positive neurons significantly increased in the “CCI” group (7491 ± 600 cells per mm^3^), in comparison to the “Sham” (1685 ± 112 cells per mm^3^, *p* < 0.001) and “Sham + Syn” (2427 ± 411 cells per mm^3^, *p* < 0.001) groups (Figure 6C,F). However, synaptamide treatment caused a significant decrease in the number of ATF3-expressing cells down to 5480 ± 697 cells per mm^3^ in the “CCI + Syn” group, in comparison to vehicle-treated rats with sciatic nerve injury (*p* < 0.05) (Figure 6C,F). Nevertheless, the number of ATF3-positive neurons in the DRG of the “CCI + Syn” group was still higher than in the ganglia of both sham-operated groups (*p* < 0.01), 14 days post-surgery (Figure 6F). At day 35 post-surgery, the number of ATF3-positive cells remained significantly elevated in both the “CCI” (3546 ± 340 cells per mm^3^) and “CCI + Syn” (3767 ± 410 cells per mm^3^) groups, in comparison to the “Sham” (1624 ± 208 cells per mm^3^, *p* < 0.01) and “Sham + Syn” (1238 ± 161 cells per mm^3^, *p* < 0.001) groups (Figure 6C,F). However, no significant difference was observed between the “CCI” and “CCI + Syn” groups at this time point (Figure 6F).

## 3. Discussion

Neuropathic pain treatment options include several recently developed alternative methods, such as using substances of natural origin, in particular, the polyunsaturated fatty acids (PUFAs) and their metabolites, which can act as neuromodulators and anti-inflammatory compounds in both peripheral and central nervous systems [6,7]. Docosahexaenoic acid (DHA) is a PUFA that exerts anti-inflammatory and neuroprotective properties [8,9,10,11]. However, DHA is believed to be active mainly through its derivatives and metabolites [12], such as neuroprotectins and resolvins [13]. Synaptamide, the compound used in the current study, is another endogenous metabolite of DHA that can promote neurite- and synaptogenesis in neuronal cell cultures [15,16,17,18] and inhibit the release of proinflammatory cytokines in microglial cell cultures after lipopolysaccharide (LPS) treatment [19,20]. We have previously discovered that in neuropathic pain conditions, synaptamide treatment results in less pronounced pain-like behavior [24,25,26], reduces inflammation in the CNS [24,26], stabilizes neurogenesis in the hippocampus [24], and increases the production of N-acylethanolamines in plasma and the brain [25]. In our current study, we examine the effects of synaptamide treatment on the peripheral nervous system after chronic constriction injury of the sciatic nerve.

Peripheral nerve injury results in the development of neuropathic pain syndrome involving spontaneous ongoing pain [1,2], the severity of which can be assessed by the weight distribution pattern between the injured and intact hind limbs [41,42,43,44]. As previously discovered, the weight-bearing deficit in the injured limb caused by chronic constriction injury of the sciatic nerve in rats peaks one week after surgery and is followed by a gradual recovery [44]. In our study, a significant weight-bearing deficit was observed in both groups of rats that underwent the CCI from the first week post-surgery. However, synaptamide treatment resulted in a markedly less pronounced weight-bearing deficit compared to the vehicle-treated animals. As shown in our previous studies [24,25,26], synaptamide treatment affects the neuropathic pain development after the CCI by decreasing mechanical [26] and thermal hyperalgesia [24], as well as cold allodynia [25,26]. The beneficial effect of synaptamide treatment on pain-like behavior is probably mediated by its anti-inflammatory properties in the peripheral nervous system.

Sciatic nerve injury is known to induce cellular and molecular changes in the peripheral nervous system, which includes the distal and proximal segments of the nerve and the dorsal root ganglia. Chronic constriction injury leads to dramatic changes to the sciatic nerve, involving demyelination and degradation of myelin sheaths, recruitment of inflammatory cells to the site of the trauma, and derangement of nerve fibers [27], all of which were observed in our study in both vehicle- and synaptamide-treated rats. In particular, demyelination after CCI is caused by the dedifferentiation of Schwann cells that form the myelin sheaths in the peripheral nerves and occurs as a consequence of axonal degeneration distal to the injury. Demyelination can be observed by the decrease in the expression of myelin markers in immunohistochemical staining [27]. According to our data, MBP-positive staining decreases drastically in the distal part of the sciatic nerve 14 days after the CCI in both synaptamide- and vehicle-treated groups. However, at day 35, synaptamide administration results in a significantly higher myelin staining area in comparison to the vehicle-treated rats. After an injury to a peripheral nerve, dedifferentiated Schwann cells form a special substrate that is permissive for nerve regeneration, and immune cells that are involved in neuroinflammation contribute to nerve restoration and remyelination via neurotrophic factor production and remove inhibitory myelin debris and toxic substances that inhibit remyelination and axonal regeneration [45]. However, unresolved neuroinflammation after peripheral nerve injury can be detrimental to the nerve structure and function [46]. 

In our previous study, we have shown that synaptamide treatment after CCI reduces inflammation in the lumbar spinal cord by downregulating iba-1-positive microglia activity, as well as IL1β concentration 35 days post-surgery [26]. Iba-1 (ionized calcium-binding adapter protein 1) is a cytoskeleton protein located in microglia and macrophages, where it acts as an actin-cross-linking protein. Iba-1 protein is involved in cell migration, membrane ruffling, and phagocytosis [47]. In the current study, at day 14 post-surgery, synaptamide administration results in a significant decrease in the activity of the CD68-positive macrophages in the distal part of the sciatic nerve in comparison to vehicle treatment. However, synaptamide does not affect iba-1- and CD163-positive macrophage activity at this time point. At the same time, 35 days after CCI, a completely different pattern of macrophage activity is observed in the sciatic nerve. In synaptamide-treated rats, CD163-positive macrophages are upregulated, whereas iba-1-positive macrophages are downregulated. However, no difference was observed in CD68-positive macrophage activity between synaptamide- and vehicle-treated rats. It is known that inflammation is regulated by the two subtypes of activated macrophages: M1- and M2-like macrophages. As previously described, CD163 is a member of the scavenger receptor cysteine-rich family and is expressed by alternatively activated anti-inflammatory M2 macrophages that promote the resolution of inflammation, angiogenesis, tissue repair, and neuropathic pain reduction [29,30]. Anti-inflammatory macrophages are also involved in nerve regeneration by removing myelin debris, expressing growth factors, and remodeling the extracellular matrix [48]. On the contrary, CD68 is a myeloid-specific surface marker that is considered a scavenger receptor type D [49] and is believed to be expressed by antigen-activated proinflammatory M1 macrophages [30]. CD68 is a heavily glycosylated type I transmembrane glycoprotein that is mainly associated with the endosomal/lysosomal compartment [50], suggesting its role in antigen processing [51]. Thus, synaptamide probably affects both the ratio of M1- and M2-type macrophages and the overall activity of macrophages at the site of nerve injury at different stages of the pathological process. 

To date, it is known that synaptamide inhibits the expression of proinflammatory cytokines by enhancing cAMP/PKA signaling and suppressing nuclear translocation of NF-κB p65 in a model of LPS-induced inflammation [22]. In our study, synaptamide treatment did not significantly affect the level of IL-1β, but markedly reduced the level of IL-6 in the distal segment of the injured sciatic nerve. As previously described, different cytokines are active at different stages of the inflammatory process [31]. IL-1β is a proinflammatory cytokine that is upregulated in the immature Schwann cells in the early stage of degeneration in the distal part of the injured nerve but decreases after the remyelination process begins [32]. Thus, the sharp decrease in IL-1β levels in both vehicle- and synaptamide-treated rats observed in our study on day 14 post-surgery might be due to the remyelination process in the injured sciatic nerve. IL-6 is a multifunctional cytokine that is upregulated in the distal part of the injury site, similar to IL-1β [34]. Schwann cells are considered the main source of IL-6 and its receptors, and its level appears higher in non-myelinating Schwann cells [34]. It has been shown that IL-6 can enhance the synthesis of myelin proteins in Schwann cells by activating the signal transducer and activator of transcription 3 (STAT3) [52] and contributes to the accumulation of macrophages in the distal segment of the injured nerve [31]. In our study, inhibition of IL-6 upregulation at day 35 occurred simultaneously with significantly increased myelin immunoreactivity in synaptamide-treated rats, which might be due to the higher rate of myelinating Schwann cells and faster remyelination process caused by synaptamide treatment. Although proinflammatory interleukins may contribute at the early stages of nerve regeneration, their prolonged overexpression may result in axonal damage and spontaneous action potential discharge in nociceptive fibers, which leads to activation of satellite glial cells and an immune response in the DRG and, subsequently, to activation of spinal microglia and production of glial-derived proinflammatory and pronociceptive mediators [53]. Thus, inhibition of IL-6 overexpression by synaptamide administration, as observed in our current study, might contribute to neuropathic pain attenuation.

Moreover, we observed that synaptamide treatment affected ATF3 activity in the primary sensory neurons in the dorsal root ganglia. ATF3 is an injury-related pro-regenerative transcription factor that, upon activation, leads to changes in gene expression in the injured neurons, and thus contributes to nerve regeneration by stimulating the intrinsic growth state of injured neurons [40]. According to our data, at day 14 after CCI the number of ATF3-positive neurons in the DRG decreases following the synaptamide administration, in comparison to vehicle treatment, but remains markedly higher than in the DRG of sham-operated rats. However, 35 days post-surgery, the number of ATF3-positive neurons following synaptamide and vehicle administration appeared at the same level in rats with CCI, which was significantly higher than in sham-operated rats. The pattern of ATF3 immunoreactivity in the DRG was similar to the activity of CD68 in the distal part of the sciatic nerve. Such dynamics of ATF3 activity further confirm the positive effect of synaptamide on the preservation of nerve fibers after CCI. However, most likely, synaptamide has no direct effect on this transcription factor, and its action is mediated through anti-inflammatory activity.

In addition, synaptamide treatment affects microglia/macrophages, satellite glial cells (SGCs), and neurotransmitter systems in the DRG after the CCI. It is known that each sensory neuron within the dorsal root ganglia forms a distinct functional and morphological unit with its SGCs. Satellite glial cells possess a number of similarities to astrocytes in the CNS; they share common functions such as ion sink, insulation, and neurotransmitter recycling, as well as some immunocytochemical properties, apart from the expression of the glial fibrillary acidic protein. GFAP is the basic protein of astrocytes’ intermediate filaments; an increase in its expression level is considered to be a morpho-chemical indicator of astroglial activation in the CNS [54], since astrocytes are known to elongate and thicken their processes upon activation [55]. Hence, GFAP is a significant marker of astrocytes that can be detected by immunohistochemistry even in a resting state in astrocytes, but not in SGCs. Nevertheless, after peripheral nerve injury, GFAP expression increases in SGCs in the ipsilateral DRG, similar to GFAP upregulation in astrocytes in the spinal cord, and can be observed via immunohistochemistry [36]. The increase in GFAP expression in astrocytes is suggested to happen as the result of neuronal activity; the same might be true for GFAP expression in SGCs, indicating a crucial role for GFAP in neuro-glial interaction [56,57]. According to our data, 14 days after sciatic nerve injury, synaptamide treatment inhibits the sharp increase in the number of SGCs around small, medium-sized, and large neurons in the DRG. However, synaptamide administration does not affect GFAP immunoreactivity at this timepoint, as its staining area did not differ significantly between the “CCI” and “CCI + Syn” groups. Interestingly, 35 days post-surgery, the number of SGCs around large and medium-sized neurons decreases even further in the DRGs of synaptamide-treated rats and becomes significantly lower than in sham-operated rats. At the same time, GFAP immunoreactivity decreases following synaptamide administration, in comparison to vehicle-treated animals, although it remains markedly higher than in sham-operated rats. Supposedly, synaptamide treatment has a significant effect on the proliferation of satellite glial cells but has a low influence on GFAP expression in the subacute period after surgery. It was previously described that SGCs undergo morphological and molecular alterations after nerve injury, which include GFAP upregulation, proliferation, and an increase in SGC coupling via gap junctions [37], and may provide additional signaling after peripheral nerve injury [58]. SGCs in the dorsal root ganglia, similar to the astrocytes in the CNS, buffer the extracellular environment via potassium and calcium channels [13]. In our previous study, the incubation of astroglia cell culture with synaptamide resulted in an upregulated Ca^2+^ concentration after aspartate-induced Ca^2+^ influx, compared to the control [25]. Supposedly, an increase in Ca^2+^ concentration may occur due to an increase in the number of N-methyl-D-aspartate (NMDA) receptors on the cellular membranes of the astrocytes [59].

In addition to the pronounced activity of synaptamide regarding satellite glial cells, synaptamide treatment significantly decreased the number of nNOS- and SP-positive neurons in the DRG at day 35 post-surgery. Substance P and NO are the most important mediators of neuropathic pain and are involved in the manifestation of allodynia and hyperalgesia, which are typical for neuropathic pain syndrome [38,60,61]. In particular, Substance P is an 11-amino acid neuropeptide that is mostly secreted by neurons and is involved in nociception and neurogenic inflammation. SP can activate the neurokinin-1 receptor (NK1R) [62] and transmit nociceptive signals via primary afferent fibers to second-order neurons of the spinal cord and brainstem [63]. In cases of peripheral nerve injury and subsequent inflammation, SP is released from primary afferent fibers, which causes upregulation of NK1R in dorsal horn neurons. NK1R coupling with phospholipase C leads to the generation of intracellular messengers and, consequently, to the depolarization of the cell membrane and stimulation of α-amino-3-hydroxy-5-methyl-4-isoxazolepropionic acid (AMPA) and NMDA receptors, which in turn, control the expression of cytokines, chemokines, and transcription factors, in particular, the nuclear factor kappa-light-chain-enhancer of activated B cells (NF-kB) [64]. NF-kB increases the synthesis of pro-inflammatory factors, including cytokines, prostaglandins, and NO, which contribute to the development of neuropathic pain and its manifestations, such as hyperalgesia [65]. Moreover, activation of nociceptive afferents after injury to the peripheral nerve may lead to increased excitability of neurons in the spinal cord, leading to the activation of NMDA receptors and the production of NO [66]. NO acts as a neurotransmitter or intercellular messenger that, in high concentrations, exhibits cytotoxicity and contributes to neuropathic pain manifestation. Moreover, the overproduction of NO by the neuronal NO-synthase is considered to be one of the fundamental causes of neuropathic pain development [39]. Thus, inhibition of SP- and NO-ergic transmission is believed to play a key role in neuropathic pain attenuation [64,67]. As shown in the current study, inhibition of NO- and SP-ergic transmission and neuropathic pain attenuation in the synaptamide-treated rats occur simultaneously with reduced inflammation and inhibition of a sharp increase in the neuro-glial index DRG. The data obtained in our current study are consistent with those obtained earlier, in which synaptamide treatment after CCI reduces the activity of SP-positive fibers and the number of nNOS-positive neurons in the lumbar spinal cord on day 35 after surgery [26].

In general, the data collected in the current study show that synaptamide has a complex effect on the post-injury processes in the peripheral nervous system. Considering the anti-inflammatory properties of synaptamide in the central nervous system shown in our previous studies [20,24,25,26], and our current data, synaptamide appears as a promising compound for the treatment of neuropathic pain. However, the mechanisms of synaptamide activity concerning neuroprotection and regeneration require additional detailed research. For future studies, the effect of synaptamide on the acute phase of neuropathic pain development should be examined in both the peripheral and central nervous systems, as well as its definitive metabolic pathways, functional properties, and mechanism of action. These findings, combined with the data collected in all previous studies on synaptamide, are required to ensure its implementation in clinical practice for the treatment of peripheral nervous system injury.

This study presents some limitations. The effect of synaptamide administration during the acute phase of sciatic nerve chronic constriction injury has not yet been examined. The study was carried out on male rats only; the effect of synaptamide administration in neuropathic pain conditions on female specimens concerning menstrual cycles has yet to be studied.

## 4. Materials and Methods

### 4.1. Drug

**Figure 7:**
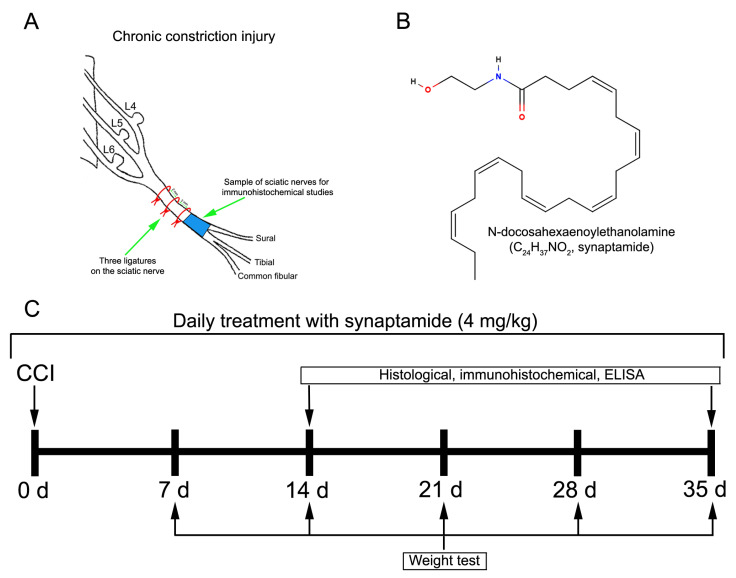
(**A**) Chronic constriction injury of the sciatic nerve. (**B**) Chemical structure of N-docosahexaenoylethanolamine (DHEA, synaptamide). (**C**) Timeline of the experiments.

The concentrate of N-docosahexaenoylethanolamine (DHEA, synaptamide) (Figure 7B) with a 99.4% purity rate was obtained as previously described by Latyshev et al. [68]. First, the liver tissue of the squid *Berryteuthis magister* was used to obtain the concentrate of PUFAs, which was later converted into ethyl esters and treated with ethanolamine (United States, Patent 3,257,436). Then, high-performance liquid chromatography on a preparative reverse-phase column Supelco Discovery HS C-18 (Sigma-Aldrich, Bellefonte, PA, USA), 10 µm particle size, 250 mm × 50 mm i.d., was performed on a Shimadzu LC-8A chromatograph (Shimadzu, Kyoto, Japan) with UV/VIS SPD-20A (205 nm), using the isocratic elution with 50 mL/min rate and the system ethanol/water (70:30, *v*/*v*), to separate fractions containing synaptamide from the ethanolamines concentrate. Synaptamide fractions were then analyzed by gas chromatography and gas chromatography–mass spectrometry after evaporation under a vacuum.

### 4.2. Animals

The experiments involved male Wistar rats (240 ± 20 g, age 3 months, n = 112) that were raised at the A.V. Zhirmunsky National Scientific Center of Marine Biology, Far Eastern Branch, Russian Academy of Sciences, Vladivostok, Russia. The rats were housed 3–4 per cage at a constant temperature (23 ± 2 °C) and humidity (55 ± 15%) on a daily 12-h light/dark cycle, with free access to food and water. All procedures were approved by the Animal Ethics Committee at the A.V. Zhirmunsky National Scientific Center of Marine Biology, Far Eastern Branch, Russian Academy of Sciences (No. 3-221019 from 22 October 2019), according to the international regulations of the European Directive 2010/63/EU and ethical guidelines for the study of experimental pain in conscious animals by the International Association for the Study of Pain.

### 4.3. Surgery

Chronic constriction injury of the sciatic nerve was used as a model of neuropathic pain, as originally described by Bennett and Xie [69]. Before the surgical procedure, all experimental rats (n = 112) were randomly divided into 4 groups: “Sham”—sham-operated animals that received vehicle treatment (n = 28); “Sham + Syn”—sham-operated rats that underwent synaptamide administration (n = 28); “CCI”—animals with chronic constriction injury of the sciatic nerve that received vehicle treatment (n = 28); “CCI + Syn”—synaptamide-treated rats with chronic constriction injury of the sciatic nerve that underwent synaptamide treatment (n = 28). For the surgical procedure, rats were subjected to general anesthesia with 4.5% isofluorane (Laboratories Karizoo, SA, Barcelona, Spain) in 100% oxygen (VetFlo, Kent Scientific Corporation, Torrington, CT, USA) in an acrylic chamber for approximately 1–2 min. During the procedure, anesthesia was maintained via a nose cone to minimize suffering [70]. For the “CCI” and “CCI + Syn” groups, after exposure, three ligatures (silk, Ethicon, Somerville, NJ, USA) approximately 2 mm apart from each other were placed on the right sciatic nerve at mid-thigh level (Figure 7A). The ligatures were gradually tightened until a slight twitching of the limb occurred. For the “Sham” and “Sham + Syn” groups, the sciatic nerve was exposed at the mid-thigh level; however, ligatures were not placed on the nerve. The skin was then sutured and treated with an antibacterial spray. Immediately after the surgery, each rat was injected subcutaneously into the loose skin over the neck with 0.9% saline (“Sham” and “CCI” groups) or synaptamide emulsion (“Sham + Syn” and “CCI + Syn”) at a dose of 4 mg/kg, which was prepared by adding saline to the synaptamide concentrate with constant shaking. Following the injection, rats were placed in home cages, with free access to food and water. The drugs were administered to experimental animals daily for 14 or 35 days after surgery (Figure 7C). 

### 4.4. Evaluation of Weight-Bearing Deficit

The severity of pain in the injured hind limb was evaluated by the weight-bearing difference test, as previously described by Nakazato-Imasato and Kurebayashi [44]. Briefly, rats (n = 28 per group) were placed into the incapacitance tester (Columbus Instruments, Columbus, OH, USA), which consisted of a plexiglass chamber with two separate sensor panels that independently measured the weight distributed by each hind paw. The pressure exerted by each paw on a sensor panel was then measured in grams within 3 s. Each rat was tested three times at 5 min intervals, during which the incapacitance tester was thoroughly cleaned with 70% ethanol to minimize olfactory signals. The three readings were then averaged to represent the weight exerted by each hind limb. The weight distribution between the right and left paw was then calculated as a percentage of the total body weight. The tests were performed weekly during the light cycle between 7:00 a.m. and 7:00 p.m. (Figure 7C). 

### 4.5. Histological and Immunohistochemical Studies

Experimental rats were sacrificed on day 14 (n = 56) or 35 (n = 56) after the chronic constriction injury (Figure 7C). The surgical procedures began only after reaching the stage of deep anesthesia in the animal, which was achieved by 4.5% isofluorane (Laboratories Karizoo, SA, Barcelona, Spain) in 100% oxygen (VetFlo, Kent Scientific Corporation, Torrington, CT, USA), with the further application via a nose cone [70]. Rats (n = 7 animals/group at each timepoint) were transcardially perfused with 200 mL of 0.1 M PBS (4 °C), pH 7.2, followed by 200 mL of 4% paraformaldehyde in 0.1 M PBS, pH 7.2 (4 °C). For histological and immunohistochemical studies, the samples of sciatic nerves 1–2 mm distal to the site of ligation and the L4–L6 ipsilateral DRG were immediately extracted and post-fixed for 24 h at 4 °C in fresh buffered 4% paraformaldehyde. 

After processing and embedding in paraffin, the 7 μm slices of the samples were prepared on a Leica rotary microtome RM 2245 (Leica, Wetzlar, Germany). For the histological examination, following deparaffinization, the longitudinal slices of the sciatic nerve were stained with hematoxylin–eosin, and the DRG slices were stained with toluidine blue (BioOptica, V2300-05, Italy). The slices were then dehydrated and embedded in a mounting medium (CS705, Dako, Santa Clara, CA, USA) for further examination.

For the immunohistochemical studies, the longitudinal slices of the sciatic nerve (for CD163, CD68, and iba-1 staining), the transverse slices of the sciatic nerve (for MBP staining), and the slices of DRG were deparaffinized and incubated in a 3% hydrogen peroxide solution to block endogenous peroxidase activity. The slices were then washed from H_2_O_2_ in 0.1 M phosphate buffer (pH 7.2), and the non-specific binding of antibodies was blocked by a 60 min-long incubation in a 2% bovine serum albumin solution (Sigma-Aldrich, Darmstadt, Germany) with 0.25% Triton X-100 (Sigma-Aldrich, Darmstadt, Germany). Subsequently, the sections were incubated with primary antibodies for 24 h at 4 °C. In particular, the sections of DRG were stained with mouse monoclonal antibodies to GFAP (1:2000, AMAB91033, Sigma-Aldrich, Darmstadt, Germany); iba-1 (1:1000, ab178846, Abcam, Cambridge, UK); substance P (1:1000, ab67006, Abcam, Cambridge, UK); nNOS (1:1000, ab40662, Abcam, Cambridge, UK), CD163 (1:500, ab182422, Abcam, Cambridge, UK); and ATF3 (1:700, HPA001562, Sigma-Aldrich, Darmstadt, Germany). The sections of the sciatic nerve were incubated with mouse monoclonal antibodies to MBP (1:1000, ab62231, Abcam, Cambridge, UK); rabbit mono- and polyclonal antibodies to iba-1 (1:1000, ab178846, Abcam, Cambridge, UK); CD163 (1:500, ab182422, Abcam, Cambridge, UK); CD68 (1:1000, ab125212, Abcam, Cambridge, UK). Negative control with no primary antibodies was also performed. Three portions of 0.1 M phosphate buffer (pH 7.2) were used to wash the slices after primary antibodies. Each section was then treated with appropriate secondary antibodies labeled with horseradish peroxidase (PI-1000, anti-rabbit; PI-2000, anti-mouse, 1:100, Vector Laboratories, San Francisco, CA, USA) for 30 min. Following three washes in 0.1 M phosphate buffer (pH 7.2), the slices were stained with chromogen (ab64238, Abcam, Cambridge, UK) for 10 min to elicit the immunoperoxidase reaction. After washing and dehydrating, the slices were embedded in a mounting medium (CS705, Dako, Santa Clara, CA, USA) for further examination.

### 4.6. Image Analysis

For the image analysis, photographs of the slices after histological and immunohistochemical staining (at least 70 per group) were captured with a CCD camera, AxioCam HRc (Carl Zeiss, Oberkochen, Germany), on a microscope, Axio Image Z2 (Carl Zeiss, Oberkochen, Germany). On each photograph, immunoreactivity in the DRG and sciatic nerve was assessed using ImageJ software (NIH, Bethesda, MD, USA). Neuro-glial index on the toluidine blue-stained slices of the DRG was counted using the Cell Counter plugin. The Cell Counter plugin was also used to calculate the number of nNOS-, SP-, and ATF3-positive cells per section within the area of interest in the DRG and the number of CD163-positive cells per section within the area of interest in the sciatic nerve. The number of cells per mm^3^ was then calculated using the formula:

$$d=\frac{1,000,000,000\times n}{7\times S}$$

where d—the number of cells per mm^3^; n—the number of cells within the area of interest on a section; S—the area of interest on a section in µm^2^; 7—the thickness of the slices in µm; 1,000,000,000—conversion factor for µm^3^ and mm^3^.

The IHC Toolbox plugin was used to evaluate the area fraction of the iba-1-, MBP-, and CD68-positive staining areas in the sciatic nerve slices and the GFAP-, CD163-, and iba-1-positive staining areas in the DRG. For the staining area evaluation, visually selected pixels were used for the statistical color detection model, which was later used for the background elimination. After binarization, the area of interest was selected for each photograph, and the area fraction was measured and expressed as the percentage of the colored area on the total area of interest.

### 4.7. Enzyme-Linked Immunosorbent Assay 

The concentration of IL-1β and IL-6 in the samples of sciatic nerves of the experimental rats (n = 7 animals/group) was determined by ELISA. First, the extraction buffer (100 mM Tris, pH 7.4, 150 mM NaCl, 1 mM EGTA, 1 mM EDTA, 1% Triton X-100, and 0.5% sodium deoxycholate), containing 1 mg/mL of protease inhibitor cocktail (Complete, Sigma-Aldrich, Darmstadt, Germany) and 0.01 mg/mL of phosphatase inhibitor cocktail (P5726, Sigma-Aldrich, Darmstadt, Germany) on ice, was used for homogenization of the samples. The rat ELISA kits for IL-1β (ab255730, Abcam, Cambridge, UK) and IL-6 (ab234570, Abcam, Cambridge, UK) were used to evaluate the cytokine concentrations in the sciatic nerve samples. A BCA protein assay kit (Pierce, Rockford, IL, USA) was then used to assess the total protein concentrations. iMark microplate absorbance reader (Bio-Rad, Hercules, CA, USA) was used to measure light absorbance at 450 nm wavelength.

### 4.8. Statistical Analysis

The datasets obtained from the weight-bearing test (n = 28 animals/group) were subjected to statistical analysis with the Mann–Whitney test and *p* < 0.05 was taken as statistically significant. The datasets obtained from ELISA (n = 7 animals/group at each timepoint), toluidine blue, and the immunohistochemical studies (n = 7 animals/group at each timepoint) were compared using one-way ANOVA tests followed by a post hoc Tukey’s multiple comparison test. All data were analyzed using GraphPad Prism 4 (GraphPad Software, San Diego, CA, USA) and shown as mean ± SEM.

## Data Availability

The datasets generated during the current study are available from the corresponding author on reasonable request.

## Abbreviations

AMPA	α-amino-3-hydroxy-5-methyl-4-isoxazolepropionic acid
ANOVA	Analysis of Variance
ATF3	Activating transcription factor 3
cAMP	Cyclic adenosine monophosphate
CCI	Chronic constriction injury
CNS	Central nervous system
DHA	Docosahexaenoic acid
DHEA	N-docosahexaenoylethanolamine
DRG	Dorsal root ganglia
ELISA	Enzyme-linked immunosorbent assay
GABA	Gamma-aminobutyric acid
GFAP	Glial fibrillary acidic protein
GPR110	G-protein coupled receptor 110
iba-1	Ionized calcium-binding adapter molecule 1
IFN-γ	Interferon gamma
IL-1β	Interleukin 1 beta
IL-6	Interleukin 6
LPS	Lipopolysaccharide
MBP	Myelin basic protein
NF-κB	Nuclear factor kappa-light-chain-enhancer of activated B cells
NGF	Nerve growth factor
NK1R	Neurokinin-1 receptor
NMDA	N-methyl-D-aspartate
nNOS	Neuronal nitric oxide synthase
NO	Nitric oxide
PBS	Phosphate-buffered saline
PKA	Protein kinase A
PUFA	Polyunsaturated fatty acid
SEM	Standard error of the mean
SGCs	Satellite glial cells
SOD	Superoxide dismutase
SP	Substance P
STAT3	Signal transducer and activator of transcription 3
TNF-α	Tumor necrosis factor α
